# The Stoichiometric Interaction of the Hsp90-Sgt1-Rar1 Complex by CD and SRCD Spectroscopy

**DOI:** 10.3389/fmolb.2017.00095

**Published:** 2018-01-17

**Authors:** Giuliano Siligardi, Minghao Zhang, Chrisostomos Prodromou

**Affiliations:** ^1^Diamond Light Source Ltd., Didcot, United Kingdom; ^2^Structural genomics Consortium, Nuffield Department of Clinical Medicine, University of Oxford, Oxford, United Kingdom; ^3^Genome Damage and Stability Centre, School of Life Sciences, University of Sussex, Brighton, United Kingdom

**Keywords:** Hsp90, Sgt1, Rar1, nod-like receptors, innate immunity, heat shock, co-chaperone

## Abstract

While the molecular details by which Hsp90 interacts with Sgt1 and Rar1 were previously described the exact stoichiometric complex that is formed remains elusive. Several possibilities remain that include two asymmetric complexes, Sgt1_2_-Hsp90_2_-Rar1_2_ (two molecules of Sgt1 and Rar1 and one Hsp90 dimer) or Sgt1_2_-Hsp90_2_-Rar1_1_ (with a single Rar1 molecule) and an asymmetric complex (Sgt1_1_-Hsp90_2_-Rar1_1_). The Hsp90-mediated activation of NLR receptors (Nucleotide-binding domain and Leucine-rich Repeat) in the innate immunity of both plants and animals is dependent on the co-chaperone Sgt1 and in plants on Rar1, a cysteine- and histidine-rich domain (CHORD)-containing protein. The exact stoichiometry of such a complex may have a direct impact on NLR protein oligomerization and thus ultimately on the mechanism by which NLRs are activated. CD spectroscopy was successfully used to determine the stoichiometry of a ternary protein complex among Hsp90, Sgt1, and Rar1 in the presence of excess ADP. The results indicated that a symmetric Sgt1_2_-Hsp90_2_-Rar1_1_ complex was formed that could allow two NLR molecules to simultaneously bind. The stoichiometry of this complex has implications on, and might promote, the dimerization of NLR proteins following their activation.

## Introduction

Nucleotide-binding domain and Leucine-rich Repeat containing proteins (NLR) act as intra- and extra-cellular sensors, effectors, or mediators in the innate immune system in plants and animals. These proteins therefore represent an initial switch for the induction of disease defense responses (Griebel et al., 2014; Wu et al., 2014). The proper functioning of NLR proteins is dependent on Heat shock protein 90 (Hsp90), a molecular chaperone that consists of three domains, an N-terminal ATP binding domain, a middle domain that is also essential for the hydrolysis of ATP and a C-terminal dimerization domain (Prodromou et al., 1997; Meyer et al., 2004; Ali et al., 2006; Prodromou, 2012). In order to understand the complex chaperoning, and indeed the cellular signaling from NLRs, requires that a detailed knowledge is known of the co-operative interactions that take place between Hsp90, its co-chaperones (Sgt1 and Rar1) and the NLR client protein. The set of interactions that take place in the Sgt1-Hsp90-Rar1 (Sgt1, suppressor of G2 allele of *skp1* and Rar1, required for *Mla12* resistance) complex have been structurally determined (Botër et al., 2007; Zhang et al., 2008, 2010), but the exact stoichiometry of the complex remains unknown.

Sgt1 consists of three domains, of which the N-terminal TPR domain appears to be dispensable for innate immunity (Takahashi et al., 2003; Lee et al., 2004; Botër et al., 2007), and instead seems to be involved in an interaction with the Skp1p-Cdc53p-F box (SCF) E3 ubiquitin ligase subunit Skp1 (Catlett and Kaplan, 2006; Kadota et al., 2008). The C-terminal domain of Sgt1 is a highly conserved SGS domain (Sgt1 specific) that interacts with NLRs (Dubacq et al., 2002; Bieri et al., 2004; da Silva Correia et al., 2007), while the middle domain is a CS domain (CHORD-SGT1 domain) that is structurally related to p23/Sba1 (Dubacq et al., 2002; Garcia-Ranea et al., 2002; Zhang et al., 2008). However, these CS domains do not share a common interaction site with the N-terminal domain of Hsp90 (Botër et al., 2007; Kadota et al., 2008; Zhang et al., 2008, 2010). Whereas, p23/Sba1 interacts with the closed ATP lid conformation of Hsp90, the CS domain of Sgt1 bound to a distinct site on the Hsp90 N-terminal domain and its interaction did not influence the state of the chaperone's ATP lid. In contrast, Rar1 possesses two CHORD domains (cysteine- and histidine-rich) that bind two zinc ions each, and both CHORD domains are known to interact with the N-terminal domains of Hsp90 (Takahashi et al., 2003; Botër et al., 2007; Kadota et al., 2010; Zhang et al., 2010; Kadota and Shirasu, 2012) as well as with the CS domain of Sgt1. It appears that the CHORD I domain of Rar1 shows tighter binding to Hsp90 (Botër et al., 2007; Zhang et al., 2010). Animals also contain similar CHORD containing proteins, melusin and Chp1, although their involvement in innate immune complexes remains to be confirmed (Shirasu et al., 1999). Melusin and Chp-1 contain an additional C-terminal CS domain, which is essential but not wholly sufficient, for binding to Hsp90 (Hahn, 2005; Wu et al., 2005).

Structural and biochemical studies have shown that Rar1 promotes the ADP-bound conformation of Hsp90 (Zhang et al., 2010). The binding of the CHORD II domain of Rar1 onto the N-terminal domain of Hsp90 appears to destabilize the ATP lid of Hsp90. Specifically, it appears that Rar1 promotes an inactive ADP-bound conformation of Hsp90 that favors Sgt1 interaction, via its CS domain, with the N-terminal domain of Hsp90 (Sbroggiò et al., 2008; Zhang et al., 2010; Prodromou, 2012). Ultimately, it appears that a stable Sgt1-Hsp90-Rar1-NLR complex might be formed that is posed for molecular recognition of an infected state (Zhang et al., 2010; Prodromou, 2012).

Inactive NLR receptors are thought to exist in a metastable conformation that involves intramolecular interactions between the various domains of NLRs (Bendahmane et al., 2002; Moffett et al., 2002; Kadota et al., 2010; Feerick and McKernan, 2017), which is promoted by Sgt1 (Leister et al., 2005). It is thought that the detection of a cognate effector induces conformational changes, leading to a dissociation of the NB-ARC or NACHT domain [nucleotide binding (NB) domains] and the LRR (leucine rich repeat) domain of NLRs, that then allows the exchange of ADP for ATP in the NB domain (Sukarta et al., 2016). Once an NLR sensor is activated this often leads to oligomerization through their central NB domains (Ade et al., 2007; Danot et al., 2009), although N-terminal domains, such as coiled coil (CC) and Toll-interleukin 1 (IL-1) receptor (TIR) domains have also been shown to drive dimerization (Inohara et al., 2000; Mestre and Baulcombe, 2006; Kadota et al., 2010; Bernoux et al., 2011; Maekawa et al., 2011; Takken and Goverse, 2012; Huber et al., 2015). In animals and plants, there is also evidence for the formation of functional pairs of different NLRs (Sinapidou et al., 2004; Ashikawa et al., 2008; Eitas et al., 2008; Lightfield et al., 2008, 2011; Birker et al., 2009; Lee et al., 2009; Eitas and Dangl, 2010; Kofoed and Vance, 2011; Okuyama et al., 2011; Halff et al., 2012; Kanzaki et al., 2012; Cesari et al., 2013; Kawano and Shimamoto, 2013; Zhai et al., 2014; Zhang et al., 2017). However, oligomerization of NLR receptors appears to be a central component in the activation of the innate immune response and the exact stoichiometric makeup of the Sgt1-Hsp90-Rar1 complex is likely impact on the mechanism of NLR oligomerization.

To date it remains an open question as to whether one or two Rar1 molecules are bound in a Sgt1-Hsp90-Rar1 complex. In turn, this could influence not only the number of Sgt1 molecules bound but also the number of NLR receptors in the complex. This ultimately could regulate the exact NLR pairs that form following their activation. Several models that differ in the number of Sgt1 and Rar1 molecules present in complex with dimeric Hsp90 are possible. These include, two symmetric complexes (Sgt1_2_-Hsp90_2_-Rar1_2_ (two molecules of Sgt1 and Rar1 and one Hsp90 dimer) or Sgt1_2_-Hsp90_2_-Rar1_1_ (with a single Rar1 molecule) and an asymmetric complex (Sgt1_1_-Hsp90_2_-Rar1_1_) that contains a single Sgt1 and Rar1 molecule in which both Rar1 CHORD domains interact with Hsp90. We therefore applied a novel circular dichroism (CD) spectroscopy method by titrating one protein component in to a preformed binary complex of two protein members of the Hsp90-Sgt1-Rar1 ternary complex in the presence of ADP. The combined analysis of the data of Sgt1 titrated on Hsp90-Rar1 complex, and subsequently Rar1 titrated on Hsp90-Sgt1 complex, revealed that a symmetric Sgt1_2_-Hsp90_2_-Rar1_1_complex was formed, containing a dimer of Hsp90 and one and two molecules of Rar1 and Sgt1, respectively. The presence of two Sgt1 molecules in the complex may promote oligomerization of NLRs and may also be directly regulated by Hsp90.

## Methods and materials

### Proteins and solution preparations

*Triticum aestivum* Hsp90, *Arabodopsis thaliana* Sgt1 and Rar1 were expressed and purified as full-length proteins as previously described (Botër et al., 2007; Kadota et al., 2008; Zhang et al., 2008, 2010).

### Far and near-UV SRCD and CD measurements

The far-UV SRCD (synchrotron radiation circular dichroism) spectra in the 185–250 nm region were measured using demountable CaF_2_ cells manufactured with recessed voids (Hellma) around the central 7 mm diameter area of 25 or 50 μm path length. The near-UV CD spectra measurements were conducted with a customized cuvette cell of 1 cm path length of 40 μl volume capacity (Hellma). The far-UV (185–250 nm) SRCD measurements diagnostic of protein folding and protein perturbations upon ligand binding were carried out using Diamond B23 beamline module station B that can penetrate much better the far UV region below 200 nm than bench-top CD instruments using a 2 mm aperture cuvette cell (Hussain et al., 2012). For the solutions investigated, the presence of 100 μM ADP in ~45 mM chloride ions in buffer containing 0.5 mM beta-mercaptoethanol (BME) and proteins at a few mg ml^−1^ concentration made the far-UV CD measurements devoid of spectral distortions unattainable using bench-top CD instruments, but possible with Diamond B23 beamline for SRCD (Hussain et al., 2012, 2015). This was not the case, however, for the near-UV region (250–330 nm) where the CD measurements, characteristic of local tertiary structure of aromatic amino acid residues and the ADP chiral adenine moiety, could be measured with the Diamond offline bench-top CD instrument Chirascan Plus using 1 s integration time and 3 nm bandwidth. All measurements were conducted at 4°C using a Peltier temperature controller (Quantum).

### Stoichiometry determination

The determination of the stoichiometry of the ternary complex using the full-length proteins Hsp90, Sgt1, and Rar1 in the presence of 100 μM ADP, which corresponded to an equivalent molar ratio of 4, was conducted as follows. First, the binding of ADP with each of the protein components of the ternary complex was investigated and then the binding of Sgt1 with Rar1 was assessed in both far and near UV regions. Since Hsp90 has been found to interact with co-chaperons and client proteins as a homodimer, for the characterization of the ternary complex two titrations were conducted. The first measured the SRCD and CD spectra for Sgt1 titrated into the preformed mixture of 50 μM Hsp90 with 25 μM Rar1 {[Hsp90:Rar1] (2:1)} leading to Hsp90:Sgt1:Rar1 (2:X:1) and the other with Rar1 titrated into the preformed mixture of 50 μM Hsp90 with 50 μM Sgt11 {[Hsp90:Sgt1] (2:2)} leading to Hsp90:Sgt1:Rar1 (2:2:Y).

For each ligand, the titrations were conducted following two consecutive methods. In the first method, the titrations in both far and near UV regions were measured for each individual solution by mixing appropriate aliquots of stock solutions of the proteins, ADP, and buffer to reach the desired molar ratio, while maintaining the total volume of the mixture constant. With this method, the Sgt1 and Rar1 protein ligand titrations were measured for 1, 2, 3, and 4 ligand molar ratios to the corresponding preformed binary protein mixtures. The second method was conducted only for the near UV titrations by adding 1 μl of stock solution of Sgt1 and Rar1, respectively, which corresponded to a 0.5 equivalent molar ratio, to each of the 125 μl volumes of preformed binary complex. This second method enabled the accurate determination of the stoichiometry of the Hsp90, Sgt1, and Rar1 ternary complex in the presence of ADP by measuring 0, 0.5, 1.0, 1.5, 2.0, 2.5, 3.0, 3.5, and 4.0 ligand equivalent molar ratios added to the corresponding preformed binary protein mixtures. For the near-UV region (250–330 nm), nine consecutive repeated CD measurements at 0.5 nm intervals corresponding to a total measuring time of 54 min were necessary to obtain a good signal-to-noise ratio due to the very small intrinsic signal of these protein-protein interaction systems.

## Results

**Figure 1:**
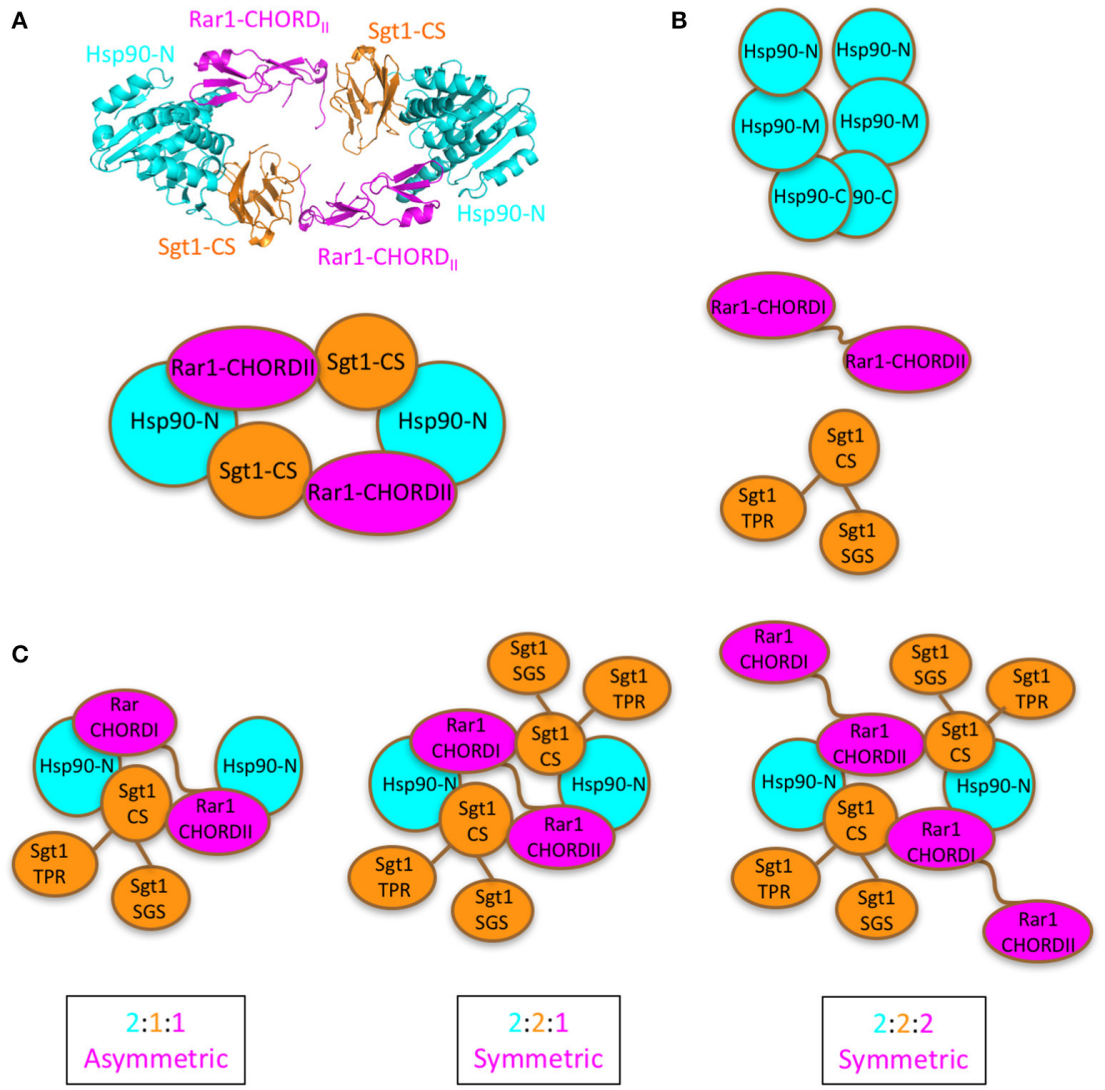
Proposed models for Sgt1-Hsp90-Rar1 complex. **(A)**, Top panel shows the crystal structure of the hetero-hexameric assembly of Hsp90-N domain (cyan), Sgt1-CS domain (gold), and Rar1-CHORDII domain (magenta) (Zhang et al., 2010). Bottom panel, cartoon of the hetero-hexameric assembly. **(B)**, Cartoon of the full-length proteins: Hsp90 with -C, -M, and -N domains (cyan), Rar1 with CHORDI-CHORDII (magenta), and Sgt1 with CS-SGS-TPR domains (gold). **(C)**, Cartoon of the possible [2:1:1], [2:2:1], and [2:2:2] stoichiometries envisaged for the full-length Hsp90, Sgt1 and Rar1 proteins as ternary complexes. For clarity the cartoons contain only the N-domain of Hsp90.

The exact stoichiometric makeup of the Hsp90-Sgt1-Rar1 ternary complex was successfully determined by CD spectroscopy by measuring the spectra in the far- and near-UV regions of the individual three protein components and their binary and ternary combinations at various molar ratios of Sgt1 and Rar1, with respect to Hsp90. The measurements of the individual proteins and the binary complexes were carried out with and without ADP. Thus, to assess the stoichiometry of the ternary protein complex we used a molar ratio of (2Hsp90:XSgt1:YRar1) in the presence of ADP (4 molar excess) to determine whether the (2:X:Y) stoichiometry was 2:1:1; 2:2:1 or 2:2:2 (Figure 1). An analogous approach, using CD spectroscopy, has been successfully applied previously to determine protein-protein interactions qualitatively, but not the stoichiometry between two co-chaperones and Hsp90 in the presence of nucleotide (Siligardi et al., 2002, 2004).

**Figure 2:**
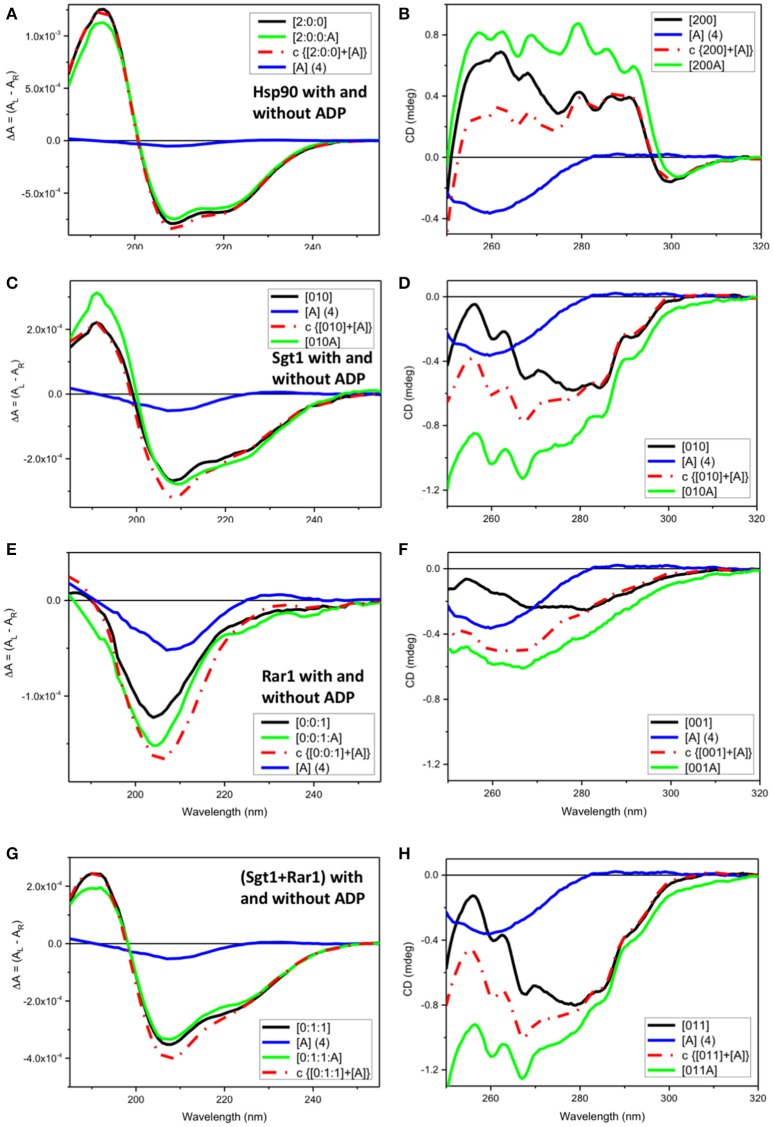
Observed SRCD and near UV CD of full-length Hsp90, Sgt1, and Rar1 with and without ADP and simulated spectra with ADP. **(A,C,E,G)** far UV SRCD and **(B,D,F,H)** near UV CD spectra. **(A,B)** Hsp90 without ADP ([200], black) and with ADP ([200A], green), calculated spectrum of Hsp90+ADP (c {[200]+[A]}, red) and spectrum of ADP ([A], blue). **(C,D)** Sgt1 without ADP ([010], black) and with ADP ([010A], green), calculated spectrum of Sgt1+ADP (c {[010]+[A]}, red) and spectrum of ADP ([A], blue). **(E,F)** Rar1 without ADP ([001], black) and with ADP ([010A], green), calculated spectrum of Rar1+ADP (c {[001]+[A]}, red) and spectrum of ADP ([A], blue). **(G,H)** Sgt1+Rar1 without ADP ([011], black) and with ADP ([011A], green), calculated spectrum of [(Sgt1)+(Rar1)]+ADP (c {[011]+[A]}, red) and spectrum of ADP ([A], blue).

For each individual protein component of the ternary complex, ADP interaction was qualitatively determined by assessing whether the observed SRCD and/or near CD spectra of the proteins with ADP were not superimposable to the simulated spectra calculated by adding together the spectra of the proteins without ADP with those of ADP of the corresponding spectral regions (Figure 2).

### Full-length Hsp90 and interaction with ADP

For full-length Hsp90 with ADP ([2:0:0:A], green), both far-UV SRCD (Figure 2A) and near-UV CD (Figure 2B) spectra were not super-imposable to the calculated spectra (c{[2:0:0]+[A]}, red) from the sum of the spectra of Hsp90 without ADP ([2:0:0], black) and that of ADP ([A] (4), blue). This was consistent with ADP binding to the Hsp90 dimer (Prodromou et al., 1997). In the far-UV SRCD region, characteristic of the protein folding, the fact that the detectable differences between the observed spectrum of Hsp90 with ADP [200A] and the calculated one (c{[200]+[A]}) were very small (Figure 2A), though detectable, indicated negligible protein conformational change in secondary structure upon binding of ADP. The difference between the observed spectrum and the calculated spectrum was much larger in the near-UV region, thus confirming the binding interaction between ADP and Hsp90.

**Figure 3:**
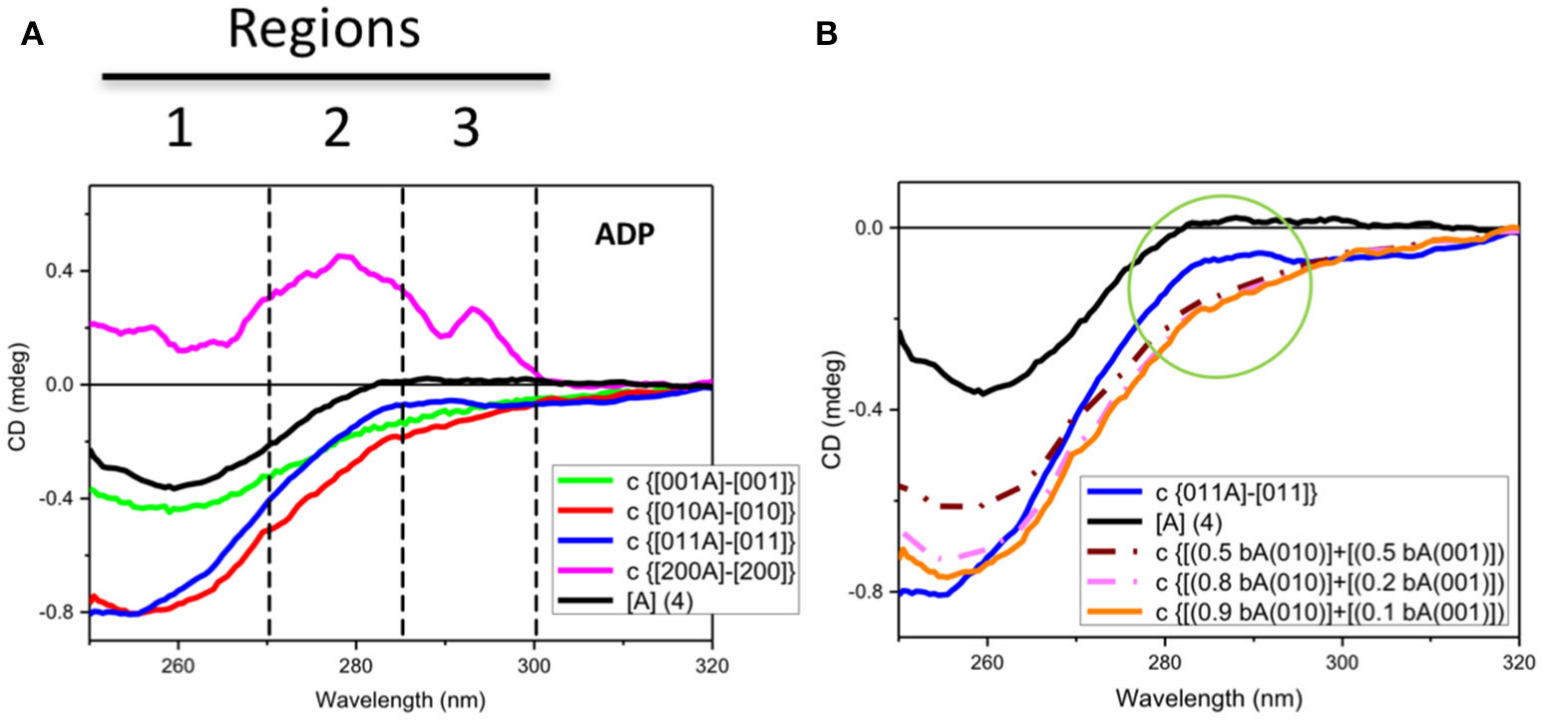
Observed and simulated CD spectra of ADP. **(A)** Observed near-UV CD spectrum of ADP ([A] (4)) (black) and the difference ADP spectra calculated by subtracting the CD spectrum of each protein without ADP to that with ADP: for Rar1 (c {[001A]-[001]} also termed bA(001) in **B**, green), Sgt1 (c {[010A]–[010]} also termed bA(010) in **B**, red), Sgt1 with Rar-1 (c {[011A]–[011]}, blue), and Hsp90 (c {[200A]–[200]}, pink). The numbers represent the molar ratios among the proteins ([1] = 25 μM). Zones 1, 2, and 3 highlight the three main CD spectral contributions that have been assigned to the protein aromatic side chains (1 for Trp and 2 for Tyr and/or Trp, and 3 for ADP and vibronic CD contributions of Phe side chain residues). **(B)** Observed near-UV CD spectrum of ADP ([A] (4)) (black) and the difference ADP spectrum for Sgt1 with Rar-1 (c {[011A]–[011]}, blue) and three simulations from linear combinations of various fractions of the difference ADP spectra, termed bound ADP (bA) for Sgt1 [010] and Rar1 [001], respectively, as calculated from Figure 4A. The simulations were calculated by adding 0.5 bA(010) to 0.5 bA(001) (brown dash-dot) and 0.8 bA(010) to 0.2 bA(001) (light pink dash-dot) and 0.9 bA(010) to 0.1 bA(001) (orange). The green circle highlights the differences between the simulated ADP spectra in the regions 2 and 3 defined in Figure 4A.

The analysis of the data conducted in a different manner revealed further details. In Figure 3, the observed spectrum of ADP was compared to the spectrum calculated by subtracting the spectrum of Hsp90 without ADP ([200]) from that of Hsp90 with ADP ([200A]). The comparison with the spectrum of ADP revealed three major features in the ADP difference spectrum that in terms of chromophore lambda maxima could not all derive from the ADP aminopurine chromophore characterized by a broad π → π^*^ electronic transition at about 260 nm (region 1 of Figure 3A). Also in region 1, distinguishable vibronic components can be seen on top of the broad transition of the adenyl group that could be due to CD contributions from phenylalanine aromatic side-chain (1^LB^) π → π^*^ transition. The positive CD band at about 280 nm was assigned to (1^LB^) π → π^*^ transition of tyrosine and/or (1^LA^) tryptophan aromatic side-chain (region 2 of Figure 3A), whereas the positive CD band at about 295 nm was assigned to tryptophan aromatic side-chain (1^LB^) π → π^*^ electronic transitions (region 3 of Figure 3A). These spectral features were better identified in the difference spectrum of ADP and were consistent with changes of the local tertiary structures of the tryptophan residues of Hsp90 that can be used to probe unambiguously the presence of molecular interactions between Hsp90 and ADP.

### Full-length Sgt1 interacts non-specifically with ADP

Both far-UV SRCD (Figure 2C) and near-UV CD (Figure 2D) spectra of Sgt1 with ADP ([010A], green) were not super-imposable to the calculated spectra (c{[010]+[A]}, red) of Sgt1 ([010], black) with ADP ([A] (4), blue) in their respective spectral regions. The SRCD and CD data analysis for Sgt1 with and without ADP indicated unambiguously that Sgt1 did interact with ADP. Sgt1 is not a known ATPase and structurally does not contain an ATP binding site. Consequently, we conclude that the interaction between ADP and Sgt1 probably represents a weak non-specific, but quantifiable and reproducible interaction under the molar equivalents used in our analysis.

### Full-length Rar1 interacts non-specifically with ADP

**Figure 4:**
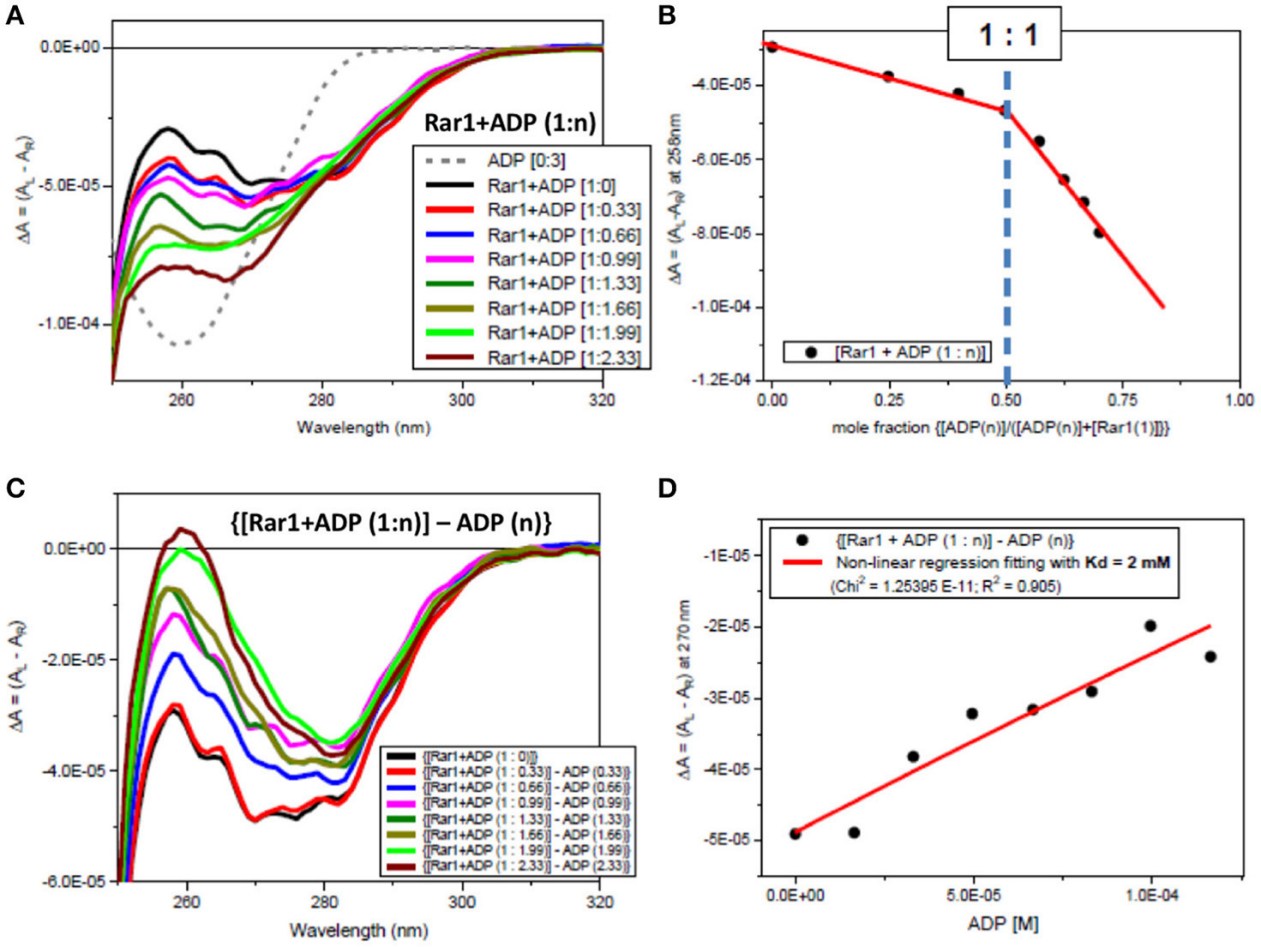
Observed CD spectra for ADP titration into Rar1. **(A)** CD spectra of the titration ADP into Rar1 at various molar ratios n: ADP 3 molar ratio (dashed gray); [Rar1+ADP (1:0)] (black); [Rar1+ADP (1:0.33)] (red); [Rar1+ADP (1:0.66)] (blue); [Rar1+ADP (1:0.99)] (magenta); [Rar1+ADP (1:1.33)] (dark green); [Rar1+ADP (1:1.66)] (dark olive); [Rar1+ADP (1:1.99)] (light green); [Rar1+ADP (1:2.33)] (brown). **(B)** Plot of ΔA intensity at 258 nm vs. mole fraction of concentration of ADP over sum of concentration of ADP plus concentration of Rar1. The plot indicates a stoichiometry of Rar1+ADP of 1:1. The thickness of the points represents the error for specific readings. **(C)** Difference CD spectra calculated by subtracting from the spectra of Rar1 with n molar of ADP the equivalent spectra of ADP of n molar ratio. **(D)** Best fitting of the CD data at 270 nm using a non-linear regression analysis described in Siligardi et al. (2002) that was calculated for a dissociation constant Kd = 2 mM. The thickness of the points represents the error for specific readings.

Both far-UV SRCD (Figure 2E) and near-UV CD spectra (Figure 2F) of Rar1 with ADP ([001A], green) were not super-imposable to the calculated spectra (c{[001]+[A]}, red) of Rar1 ([001], black) with ADP ([A] (4), blue) in their respective spectral regions. The SRCD and CD spectra of the protein Rar1 with and without ADP indicated unambiguously that Rar1 did interact with ADP. The titration of ADP with Rar1 showed a CD spectral change that was ADP concentration dependent (Figure 4A). The plot of CD intensity vs. molar fraction of {[ADP]/([ADP]+[Rar1]} showed a clear change in slope for the mole fraction of 0.5 indicative of a 1:1 binding site (Figure 4B). The apparent dissociation constant, Kd, was calculated following the same procedure developed in Siligardi et al. (2002). The CD data were transformed into difference CD by subtracting to the observed spectra of the Rar1 with ADP at various molar ratios the equivalent ADP spectra (Figure 4C). The fitting of the CD intensity at 270 nm as a function of ADP concentration using a non-linear regression analysis indicated a very weak Kd of 2 mM (Figure 4D). This represents a weak non-specific binding and is consistent with no known ATPase activity for Rar1. In Figure 3A, the comparison between the near-UV CD spectrum of ADP (black) and the difference spectrum of ADP (c{[001A]–[001]}, green) showed a very small difference in magnitude, but not in shape, that though consistent with an ADP binding interaction was ascribed mainly to the ADP adenine chromophore.

### Sgt1 and Rar1 form a binary complex in the absence of ADP

**Figure 5:**
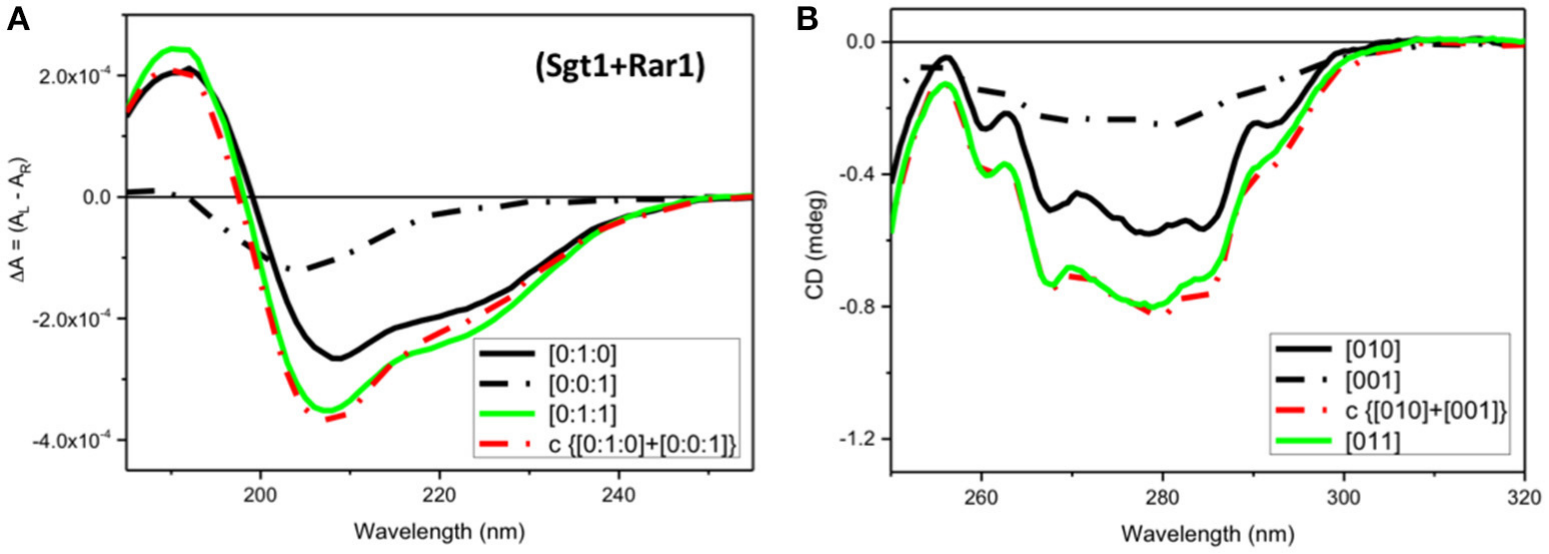
CD Spectra for Rar1-Sgt1 binary complex. Far-UV SRCD **(A)**, and near-UV CD **(B)**, spectra of Sgt1 with Rar1 [Sgt1:Rar1] at molar ratio [0:1:1] (green) and the calculated spectrum of {[0:1:0]+[0:0:1]} (red) obtained by adding the spectrum Sgt1 ([0:1:0]) (solid black) to that of Rar1 ([0:0:1]) (black, dash-dot).

In the far-UV region a very small spectral difference between the observed SRCD spectrum (Figure 5A, [011], green) and the calculated one (Figure 5A, c{[010]+[001]}, red) from the sum of the Sgt1 spectrum with that of Rar1 was indicative of minute conformational changes as the result of a binding interaction between the two full-length proteins.

In contrast, the near-UV CD spectrum of the mixture of Sgt1 with Rar1 without ADP (Figure 5B, [011], green) was identical to the calculated sum of Sgt1 spectrum with that of Rar1 (Figure 5B, c{[010]+[001]}, red) indicating that no detectable conformational change or perturbation of the local environment of the aromatic amino acid residue side-chains were observed.

### Sgt1-Rar1 complex interacts non-specifically with ADP

Both far-UV SRCD (Figure 2G) and near-UV CD spectra (Figure 2H) of the mixture of Sgt1 with Rar1 with ADP ([011A], green) were not super-imposable to the calculated spectra (c{[011]+[A]}, red) of Sgt1-Rar1 complex ([011], black) with that of ADP ([A] (4), blue). This is consistent with both Sgt1 and Rar1 interacting very weakly with ADP (Figures 2C,F).

For the mixture of Sgt1 and Rar1 (1:1), the system becomes more complicated in terms of changes of CD profiles induced by the interactions with ADP because the ADP molecules, of which the concentration has been kept constant at four molar equivalents, are distributed between the two proteins according to the ratio between the two corresponding dissociation constants. To evaluate this, a careful spectral analysis of simulated spectra was performed. In Figure 3B the calculated CD spectrum of the ADP with the mixture of Sgt1 and Rar1 (blue solid) was significantly different than those calculated from the linear combinations of various fractions (0.5+0.5, brown dash-dot), (0.8+0.2, pink dash-dot) and (0.9+0.1, orange) of the CD spectra representing ADP bound to Sgt1 and Rar1, respectively (Figure 3A). This is compelling indication for the interaction between the two Sgt1 and Rar1 full-length proteins and each protein non-specifically with ADP.

In terms of CD contributions, the calculated spectrum of ADP in the mixture of Sgt1 and Rar1 (molar ratio 1:1) (Figure 3A, c{[011A]-[011]}, blue) was similar to those interpreted for the individual Sgt1 and Rar1 proteins with ADP and that they were due to the ADP chromophore at 260 nm with negligible contributions from the proteins aromatic side-chain residues.

### Determination of the stoichiometry of the full-length protein complex

**Figure 6:**
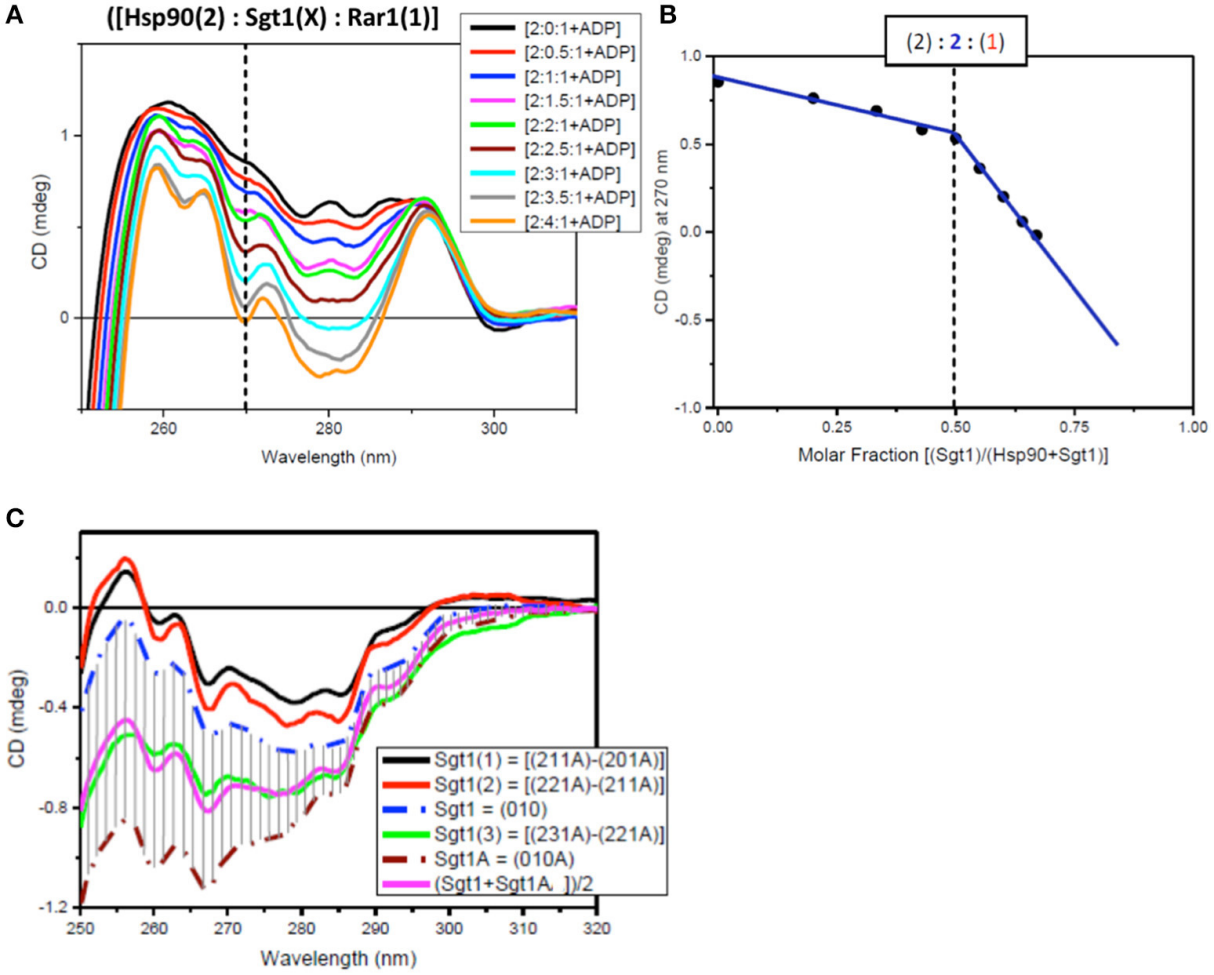
Near UV spectra for the titration of Sgt1 into a binary complex of Hsp90-Rar1 complex. **(A)** Near UV CD titration of full-length Sgt1 titrated into preformed mixture of Hsp90 with Rar-1 at [2:1] molar ratio in the presence of ATP. Sgt1 was added at consecutive additions of 0.5 molar equivalents until reaching a final 4.0 molar ratio excess (1 equivalent molar ratio = 25 μM). **(B)** Plot of CD intensity at 270 nm (dashed line) vs. mole fraction of Sgt1 calculated as follows: {[Sgt1 (X)]/([Hsp90(2)]+[Sgt1 (X)])}. The change in the slope occurring at molar fraction 0.5 indicated that the stoichiometry of the ternary complex was [2:2:1]. The thickness of the points represents the error for specific readings. **(C)** Near-UV CD spectra of Sgt1 with ADP (brown, dash-dot) and without ADP (blue, dash-dot). The solid lines are the difference CD spectra of the molar equivalent of Sgt1 titrated into the preformed [Hsp90(2):Sgt1(X):Rar1(1):ADP(4)] complexes calculated by subtracting the spectra of the complexes with one less molar increment of Sgt1, such as: {[2:1:1A]–[2:0:1A]} (black), {[2:2:1A]–[2:1:1A]} (red), and {[2:3:1A]–[2:2:1A]} (green). The pink spectrum is the linear combination of 50% of Sgt1 with ADP ([0:1:0A]) with 50% of Sgt1 without ADP ([0:1:0]).

**Figure 7:**
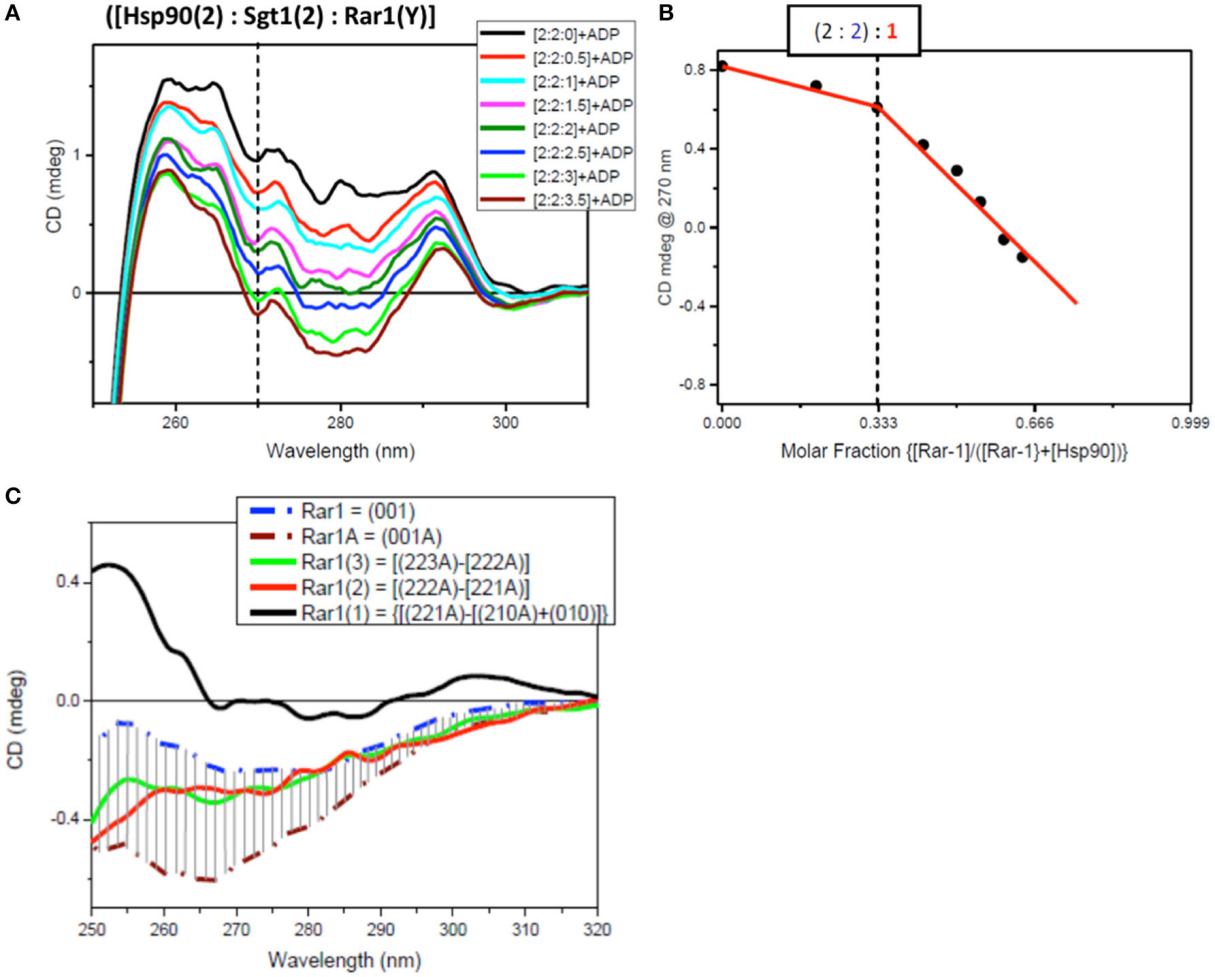
Near UV spectra for the titration of Rar1 into a binary complex of Hsp90-Sgt1 complex. **(A)** Near UV CD titration of full-length Rar1 into preformed mixture of Hsp90 with Sgt1 at [2:2] molar ratio in the presence of ATP. Rar1 was added at consecutive additions of 0.5 molar equivalent until reaching a final 3.5 molar excess. **(B)** Plot of CD intensity at 270 nm (dashed line) vs. mole fraction of Rar1 calculated as follows: {[Rar1(Y)]/([Hsp90(2)]+Rar1(Y))}. The change in the slope occurring at molar fraction 0.333 indicated that the stoichiometry of the ternary complex was [2:2:1]. **(C)** Near-UV CD spectra of Sgt1 with ADP (brown, dash-dot) and without ADP (blue, dash-dot). The solid line spectra are the difference CD spectra of the equivalent Rar1 titrated into the preformed [Hsp90(2):Sgt1(2):Rar1(Y):ADP(4)] complexes calculated by subtracting the spectra of the complexes with one less molar ratio of Rar1, such as: {[2:2:3A]–[2:2:2A]} (green), {[2:2:2A]–[2:2:1A]} (red), and {[2:2:1A]-[2:2:0A]} (black).

The determination of the stoichiometry of the full-length protein complex using Hsp90[2]:Sgt1[X]:Rar1[1]:ADP and Hsp90[2]:Sgt1[2]:Rar1[Y]:ADP involved titration of increasing amounts of Sgt1[X] and Rar1[Y] to higher molar ratios. The innovative method used to assess the stoichiometry of the ternary protein complex between Hsp90, Sgt1, and Rar1 in the presence of ADP was based on the correlation found between the results of two CD titrations in the near-UV spectral region. The first was that for Sgt1 titrated into the binary complex of Hsp90:Rar1 (molar ratio 2:1) with ADP {Hsp90(2):Rar1(1)+ADP] and the second for Rar1 titrated into the binary complex of Hsp90:Sgt1 (molar ratio 2:2) with ADP [Hsp90(2):Sgt1(2)+ADP]. The ligand proteins Sgt1 and Rar1 were both titrated into the cuvette cell containing the preformed binary complexes, with ADP, at 0.5 molar increments to reach the final molar ratio of 4 and 3.5, respectively (Figures 6A, 7A).

For the first near-UV CD titration of Sgt1 into the preformed complex, Hsp90(2)- Rar1(1) with ADP, the plot of CD intensity at 270 nm vs. the molar fraction of Sgt1, calculated as the ratio of molar concentration of Sgt1 over the sum of the concentration of Sgt1 with 50 μM Hsp90 (represented as [Hsp90(2)]), enabled the determination of the stoichiometry to be [2:2:1] rather than [2:1:1] (Figure 6B).

Another way to analyze the CD titration data was to compare the spectra of Sgt1 with and without ADP with those for the equivalents of Sgt1 calculated by subtracting from each ternary protein mixture with ADP the spectrum with the lower molar amount of Sgt1, called Sgt1(1) for {[2:1:1A]–[2:0:1:A], Sgt1(2) for {[2:2:1A]–[2:1:1:A]}, and Sgt1(3) for {[2:3:1A]–[2:2:1:A], respectively (Figure 6C). The calculated spectra of Sgt1(1) and Sgt1(2) falling outside the gray area delimited by the spectra of Sgt1 (without ADP) and Sgt1A (with ADP; Figure 6C) indicated that the calculated spectra for Sgt1(1) and Sgt1(2) were consistent with spectral changes due to binding interactions with the other two protein components, Hsp90 and Rar1 in the ternary complex. Sgt1(3) on the other hand, falling within the gray area in Figure 6C was indicative of Sgt1 binding to ADP, as saturation was reached in Hsp90:Sgt1:Rar1 complex at 2:2:1 molar ratio. The spectrum of Sgt1(3) (Figure 6C, green) was very similar to that simulated by the sum of 50% of the Sgt1 spectrum to 50% of that for Sgt1 with ADP [(Sgt1A+SgtiA)/2, pink spectrum of Figure 6C]. This is in agreement with the attributed stoichiometry of (2:2:1) for the Hsp90:Sgt1:Rar1 complex in the presence of a 4 molar ratio ADP (Figure 6B).

For the second near-UV CD titration for Rar1 into preformed binary complex Hsp90:Sgt1 (molar ratio 2:2) with ADP, the plot of the CD intensity at 270 nm vs. the molar fraction of Rar1, calculated as ratio of molar concentration of Rar1 over the sum of the concentration of Rar1 with 50 μM Hsp90 (represented as [Hsp90(2)]), confirmed the stoichiometry for Hsp90:Sgt1:Rar1 with ADP of [2:2:1+ADP] (Figure 7B) as observed in the first titration with Sgt1 (Figure 6).

The analysis of the difference CD spectra of Rar1(1), Rar1(2), and Rar1(3) calculated by subtracting the spectra of the ternary mixtures with lower molar ratio “n” of Rar1 [Hsp90(2):Sgt1(2):Rar1(n)] from that with higher “n+1” molar ratio [Hsp90(2):Sgt1(2):Rar1(n+1)] and compared to the spectra of Rar1 alone or with ADP (Rar1A; Figure 7C), showed that only Rar1(1) fell outside the demarked area between the spectra of Rar1A and Rar1 (with and without ADP, respectively). As for Sgt1, this was indicative of the binding interaction of Rar1(1) to the preformed binary complex of Hsp90:Sgt1 (2:2) (Figure 7C). The fact that the difference spectra of Rar1(2) and Rar1(3) were within the demarked area was consistent with an excess of Rar1 not interacting anymore with the complex, Hsp90:Sgt1:Rar1+ADP (2:2:1+A), but with the remaining ADP. This analysis confirmed the stoichiometry 2:2:1 for Hsp90:Sgt1:Rar1 in the presence of a 4 molar ratio of ADP as determined in Figure 7B.

## Discussion

The stoichiometry of the ternary complex of Hsp90, Sgt1, and Rar1 in the presence of ADP was assessed by CD spectroscopy. The approach used the characterization of the interactions of the individual proteins with ADP, the combination of the binary complexes and the titrations of Sgt1 into the preformed binary complex of Hsp90 with Rar1, at 2:1 molar ratio, respectively, and finally Rar1 titration into the preformed binary complex of Hsp90 with Sgt1, at a 2:2 molar ratio. The inclusion of ADP was based on previous work that showed that Rar1 could bring about the hydrolysis of ATP by Hsp90 and produce an active stable ADP-bound Rar1-Hsp90-Sgt1 complex (Zhang et al., 2010). Such long-lived stable complexes may act as potential sensors posed to respond to small molecules from invasive organisms (Prodromou, 2012).

The innovative titration method revealed that all three proteins did interact with ADP. Interaction with ADP has previously been seen with Chp1 and melusin although they have not been reported to be ATPases and these interactions probably represent some non-specific association (Hong et al., 2013). For the two titrations, both plots of the CD intensity at 270 nm vs. the mole fraction of each titrating protein, Sgt1 and Rar1, were consistent with a stoichiometry for Hsp90:Sgt1:Rar1 of (2:2:1) in the presence of a 4 molar ratio ADP (Figure 6B). We conclude that because Rar1 and Sgt1 are not known ATPases that the interaction with nucleotide is non-specific. This was demonstrated for Rar1 in which the titration with ADP revealed a very weak Kd of 2 mM (Figure 4D).

The stoichiometry of 2:2:1 (Hsp90_2_-Sgt1_2_-Rar1_1_) agrees with the symmetric model proposed in Figure 1. The model suggests that a single Rar1 molecule is bound between the two N-terminal domains of Hsp90, rather than two Rar1 molecules each interacting solely with either their CHORD I or II domains. This model is consistent with that previously proposed and with biochemical studies that suggest that the CHORD I domain of Rar1 provides the main binding affinity for Hsp90, but the CHORDII domain also interacts with HSp90 (Zhang et al., 2010). We thus conclude that Rar1 most likely bridges the two N-terminal domains of Hsp90 by interactions involving both its CHORD domains.

**Figure 8:**
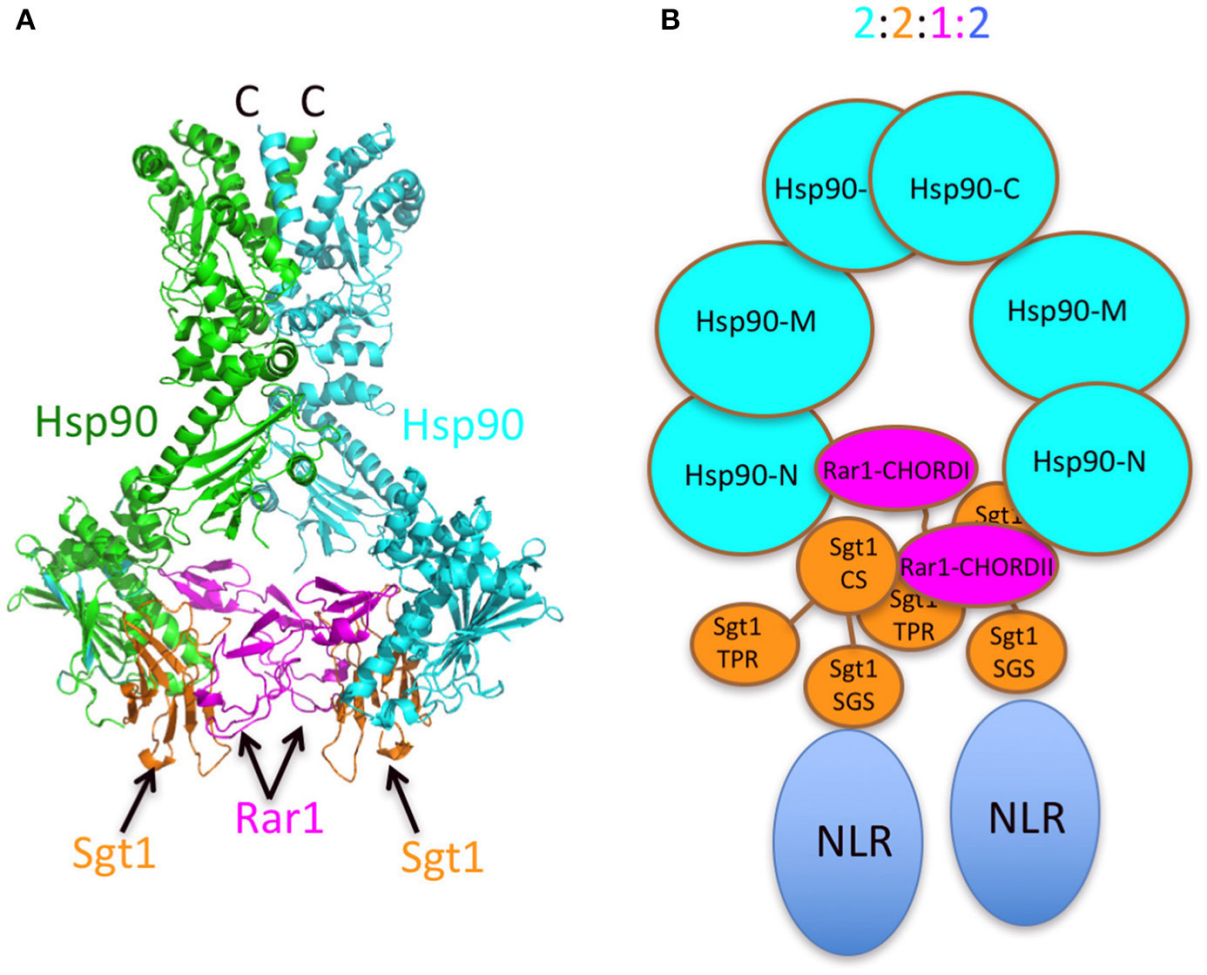
Models of the Sgt1-Hsp90-Rar1 complex. **(A)** Model of full-length Hsp90 complexed with Sgt1 and Rar1 based on known structures (Ali et al., 2006; Zhang et al., 2010). **(B)** Cartoon of the Hsp90-Sgt1-Rar1-NLR complex (2:2:1:2 molar ratio, respectively) based on the 2:2:1 stoichiometry of the Sgt1-Hsp90-Rar1 complex as determined by CD spectroscopy.

The presence of two Sgt1 molecules in the complex is consistent with observed structures (Zhang et al., 2010). The presence of two Sgt1 molecules in the Hsp90 complex raises the possibility that each could recruit either two of the same NLR or distinct NLR pairs to stably associate within the Hsp90 complex. That both Rar1 CHORD domains appear to interact with Hsp90 and their ability to interact with the CS domains of Sgt1 suggest that both Hsp90 N-terminal domains are converted to an ADP-bound state as seen in structural studies of a Rar1 CHORD II-Hsp90-Sgt1 CS domain complex. Activation of either one or both NLR proteins could then lead to a Hsp90 regulated association of the NLR proteins into an active state, thus initiating disease defense responses (Figure 8). In support of such a model, Hsp90 has been reported to be involved in the assembly of macromolecular complexes. For example, Hsp90 has been shown to mediate the assembly and nuclear import of influenza A virus RNA polymerase complex (Momose et al., 2002; Naito et al., 2007), the incorporation of small RNAs into Argonaute (Nagradova, 2002; Iki et al., 2010, 2012; Miyoshi et al., 2010; Iwakawa et al., 2012), the binding of the reverse transcriptase to pregenomic RNA templates (Kawasaki and Shimizu, 1996; Stahl et al., 2007a,b) and the assembly of the Replicase Complex of a Positive-Strand RNA Plant Virus (Mine et al., 2012). Clearly, Hsp90 can bring about macromolecular assembly and it is not unreasonable that it may have such a role in oligomerization of NLR pairs. However, Melusin and Chp1 in animals also contain a CS domain, that might modify the number of Sgt1 molecules bound in a Hsp90 complex by competing with the CS domain of Sgt1 for binding. Alternatively, the CS domain of these CHORD containing proteins might be responsible for recruitment of other as yet unknown proteins and may represent a fundamental difference between plant and animal Hsp90-NLR complexes. Thus, while there may be differences in plant and animal systems, this study forms the basis of a model, in which Hsp90 can regulate NLR oligomerization that can now be tested further.

## Author contributions

CP and GS: designed the research; GS, MZ, and CP: carried out the experimentation; CP: supervised all the work; CP and GS: wrote the paper and CP and GS: contributed to subsequent drafts.

### Conflict of interest statement

The authors declare that the research was conducted in the absence of any commercial or financial relationships that could be construed as a potential conflict of interest.
